# Neuroradiological Correlation of the Lateral Third Periventricular, Pituitary Gland and Stalk, Pineal Gland, Cerebral Aqueduct, and Foramen Magendie and Luschka With Intraventricular Neuroendoscopic Images: A Case Series

**DOI:** 10.7759/cureus.56952

**Published:** 2024-03-26

**Authors:** Zaitun Zakaria, Muhammad Ihfaz Ismail, Song Yee Ang, Zamzuri Idris

**Affiliations:** 1 Department of Neurosciences, School of Medical Sciences, Universiti Sains Malaysia (USM), Kota Bharu, MYS; 2 Department of Neurosciences, Hospital UniversitI Sains Malaysia (HUSM), Kota Bharu, MYS; 3 Department of Neurosciences, School of Medical Sciences, Hospital Universiti Sains Malaysia, Universiti Sains Malaysia (USM), Kota Bharu, MYS

**Keywords:** liliequist membrane, lamina terminalis, third ventricle, pituitary, pineal, neuroendoscopy, neuroradiology

## Abstract

Neuroendoscopy procedures in pediatrics have expanded beyond the endoscopic third ventriculostomy. As such, a direct and angled endoscope allows further visualization around the corner, capturing the surrounding anatomy. Intraoperative live images look different than radiological images. Hence, in this single institutional experience, we correlate neuroradiology images with intraoperative intraventricular endoscopic views of the third-fourth ventricle, pituitary, pineal gland, cerebral aqueduct, and foramen magendie and luschka. Our collective case series reveals a few interesting case scenarios of normal and abnormal findings during the procedure. Careful navigation of the neuroendoscope is crucial to prevent injury to the neurovascular bundle. A close relationship with normal anatomy from radiological imaging is necessary to prevent it from getting lost once inside the ventricular cavity.

## Introduction

In the last 20 years, the neuroendoscopic armamentarium has expanded, and the technique has gained interest, with many centers exploring this approach. The procedure is considered a minimally invasive surgery and an alternative to craniotomy. The field of neuroendoscopy in pediatrics has expanded, including endoscopic third ventriculostomy (ETV), washout of the intraventricular hemorrhage (IVH) or infected cerebrospinal fluid (CSF) with ventriculitis [1,2], fenestration of multiloculated cyst [3], or septum pellucidotomy [4]. The application of neuroendoscope in neurosurgery allows for real visualization of the structure within and around the ventricles. Correlating with radiological images, live images captured might appear differently. For example, magnetic resonance imaging (MRI) brain in patients with ventriculitis appears as ependymal enhancement when gadolinium material is administered. Pial or dura signal abnormality or enhancement is also detected following meningitis [5]. However, the live images will show the presence of debris and pseudomembrane on the ventricular wall. During the ETV procedure, the third ventricular floor appeared thickened and opaque. Neuroendoscopic classification of cerebral ventricular infection has previously been described by Guan et al. [6]. In addition, the intensity signal of MRI of the CSF study may not be able to confirm the normal appearance of the CSF colour (clear and colourless in normal conditions) with live images appearing as yellowish or light turbid colour when there is infective material present.

One must be mindful of the findings from radiology imaging and relate those images with what should be expected during the surgery [7]. Despite having a panoramic intraventricular view, the space is limited by the small skin incision (usually a single burr hole) and requires careful manipulation of a neuroendoscope. Hence, an intimate relationship between those images makes the surgeon safer during surgery. This article shares some of the illustrative collections of neuroendoscopic images with a comparison to radiological imaging.

## Materials and methods

The patient’s clinical history, neuroendoscopic images, and radiological imaging were retrospectively taken from selected case series to depict and correlate the endoscopic images with radiological imaging. These include pediatric cases ranging from infants to children. Preoperative brain MRI brain was performed in most cases, emphasizing constructive interference in steady state (CISS) sequence and postgadolinium, except in emergency cases. Hence, pre- and postcontrast brain computed tomography (CT) was sufficient. A 30-degree intraoperative neuroendoscope (Aesculap Inc., Hazelwood, MO) was used in most cases or an endoscope system (Carl Zeiss Meditec, Germany) when available. Basically, the surgery requires two surgeons, a primary surgeon and an assistant to hold the lower part of the neuroendoscope close to the bony surface. The ETV entry site is at the precoronal suture or 1 cm anterior to the coronal suture and should be as close to the midline as possible (normally 2-2.5 cm from the midline). For the creation of a stoma, bipolar coagulation is applied to roughen the surface and then perforated and dilated using a French Fogarty catheter to a maximum of 10 mm. To reach the pineal or posterior third of the third ventricle, the burr hole is more anterior (normally 3-5 cm anterior to the coronal suture). Image-guided surgery (IGS) (StealthStation™ S8 Cranial Solution; Medtronic, Louisville, CO) was used to present the image data directly on the patient, especially during the endoscopic fenestration of the intraventricular cysts or when closed to the pineal region or neurovascular structures. For the cyst fenestration, a "hook technique" is used whereby the membrane is hooked with forceps and cut with scissors. Bipolar coagulation may also be used to remove the arachnoid layer away from the surgery site. For the wound closure, the dura and cranial fascia are approximated with the “tight closure” technique using 4/0 prolene.

## Results

Appreciation of the fornices, thalamus, and striatum

**Figure 1:**
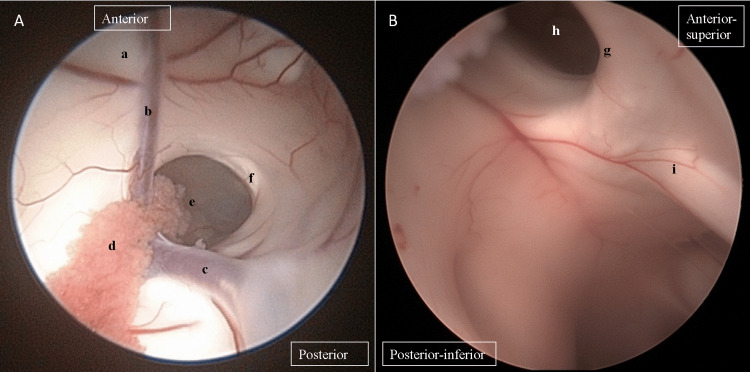
The neuroendoscopic images at the foramen of Monro. (A) The visualized images when the neuroendoscope is advanced via the right frontal horn. (B) The visualized images at the margin of the right foramen of Monro. a, septum; b, anterior septal vein; c, right thalamostriate vein; d, choroid plexus; e, right foramen of Monro; f, right fornix; g, left fornix; h, left foramen of monro; i, anterior commissure.

**Figure 2:**
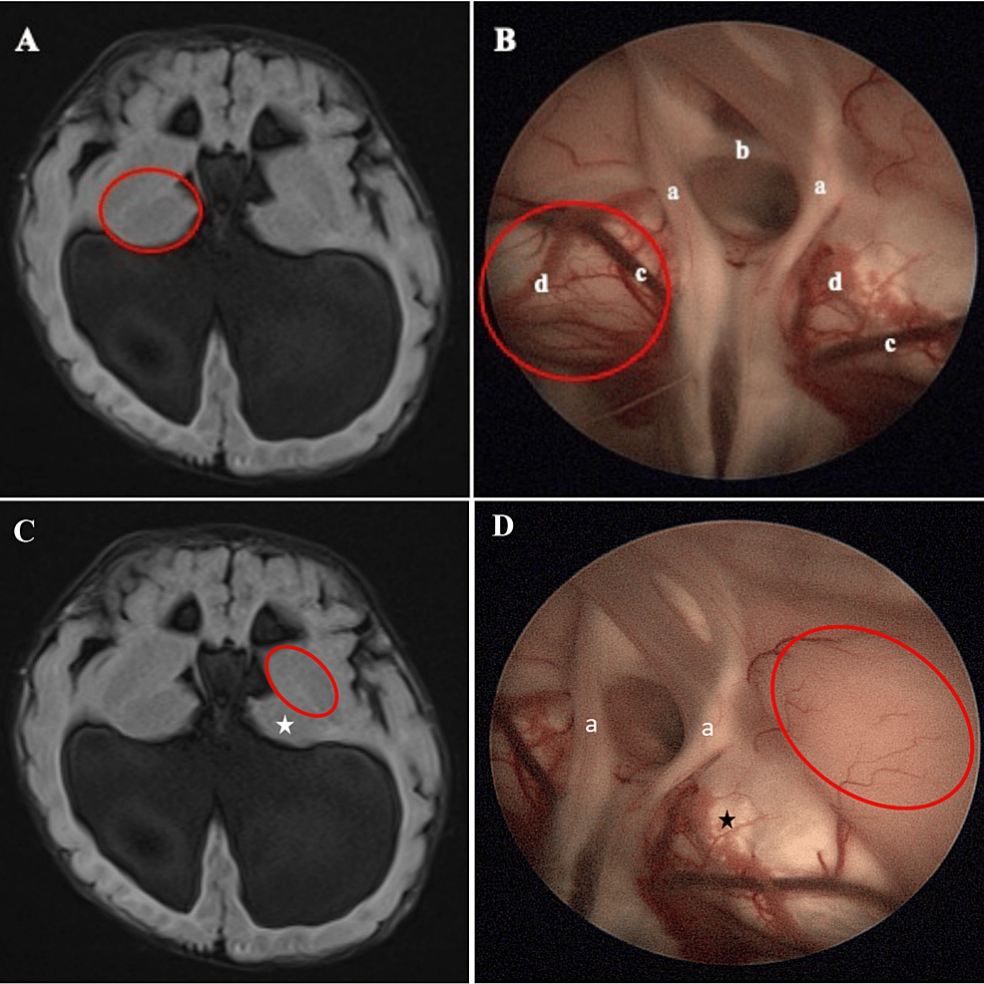
A 6-year-old child with a known Chiari malformation presented with raised intracranial pressure. She was subjected to an endoscopic third ventriculostomy (ETV). The radiology images are of an axial FLAIR MRI brain. (A, B) Due to the absence of septum pellucidum, the endoscope captured the anatomical view on both sides of the hemisphere. The red circle is an area of the thalamus that appears yellowish. (C, D) However, further antero-superior-lateral, the striatum appears slightly brownish (red circle). Star, thalamus; a, bilateral fornices; b, anterior commissure; c, thalamostriate vein; d, choroid plexus.

The usual entry into the third ventricular floor is via the foramen of Monro, with the entry site through the right or left frontal horn (Figure 1). The bulk of the choroid plexus should be recognizable, i.e., anatomically at the medial side of the foramen of Monro, passing into the third ventricle. However, when the septum pellucidum is absent, the endoscopic view will demonstrate bilateral anatomical structures. Figure 2 depicts images of a child presented with raised intracranial pressure (ICP) and Chiari malformation. An ETV procedure was performed before further surgical intervention. The fornices, bilateral thalami, and striatum [8] can be visualized. Despite the similar intensities of the thalami and striatum from a radiological perspective, their real images are contradictory.

The anterior segment and floor of the third ventricle in a child without and with a history of ventriculitis

**Figure 3:**
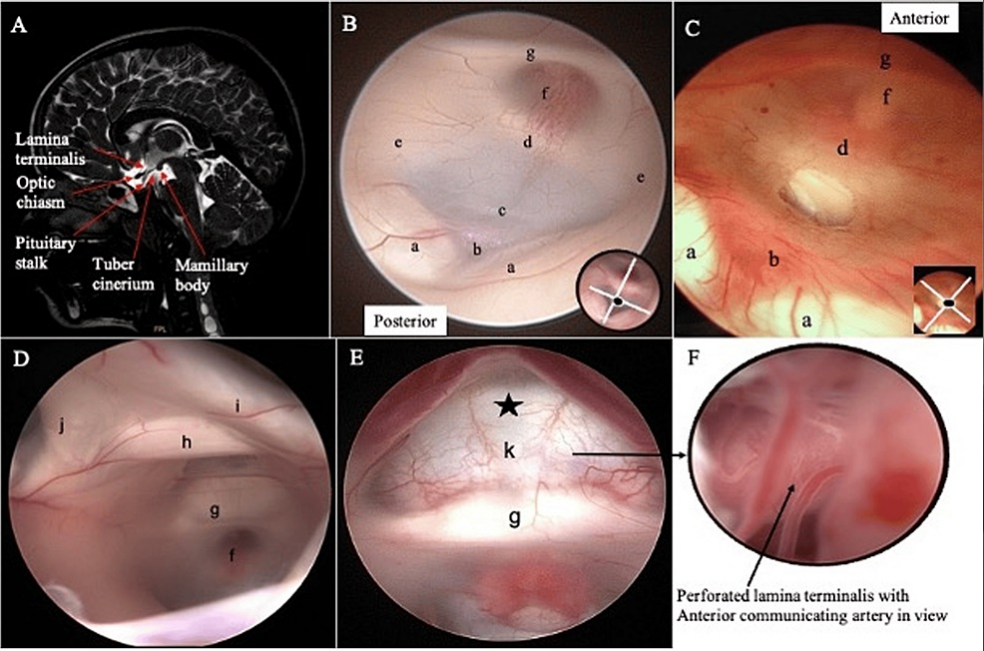
Anatomical images of the floor and anterior part of the third ventricle. (A) Mid-sagittal CSF DRIVE MRI brain shows the anatomical structure at the region of the third ventricle. (B) In a normal ventricle, the floor of the third ventricle is whitish. (C) However, in a child with ventriculitis, the floor appears thick and reddish, and the inflammatory reaction makes it difficult to appreciate the adjacent anatomy (the image shows that the stoma is already created during the ETV procedure). (D) An angled neuroendoscope is used to view the anterior part of the third ventricle. (E) The lamina terminalis, a thin white matter membrane, appears intact. (F) In the case of perforated lamina terminalis, the anterior communicating artery can be seen. a, mamillary bodies; b, basilar artery underneath; c, the floor of the third ventricle; d, dorsum sella; e, hypothalamus; f, infundibular recess; g, elevation secondary to optic chiasm; h, anterior commissure; i, right fornix; j, left fornix; k and star, intact and thin lamina terminalis (can see the dura at the base).

The endoscopic third ventriculostomy procedure is frequently used in treating pediatric patients with hydrocephalus. The technique is commonly advocated in a non-communicating (obstructive) hydrocephalus, allowing free drainage of CSF into spaces where it can be naturally absorbed. The indication is either as the primary procedure or in place of a shunt revision for shunt malfunction (secondary ETV) [9]. Furthermore, recent analysis supports a greater benefit achieved using ETV compared to the ventriculoperitoneal shunt (VPS) procedure in both the incidence of complications and mortality [10]. The site for the ETV procedure is the distance between the infundibular recess and mamillary bodies, with a mean of 6 mm (range: 3.5-9 mm) [11]. Hence, an ETV procedure is, in principle, the creation of a ventriculocisternostomy or stoma on the floor of the third ventricle (Figure 3).

Ventriculitis, an inflammation of the ependymal lining of the cerebral ventricles, is a dreaded complication from either poorly treated blood-borne infection, such as meningitis and brain abscess, or various neurosurgical procedures, including ventricular catheter-related infections and ETV [5,12]. Ventriculitis is commonly associated with communicating hydrocephalus, as the debris from infective materials blocks the ependymal layer, leading to impaired reabsorption of CSF [13]. This causes a thickened third ventricular floor, and the inflammatory reactions make it difficult to see the normal anatomical structure. In this case, a navigated image-guided surgery (IGS) is helpful.

The pituitary stalk and gland

**Figure 4:**
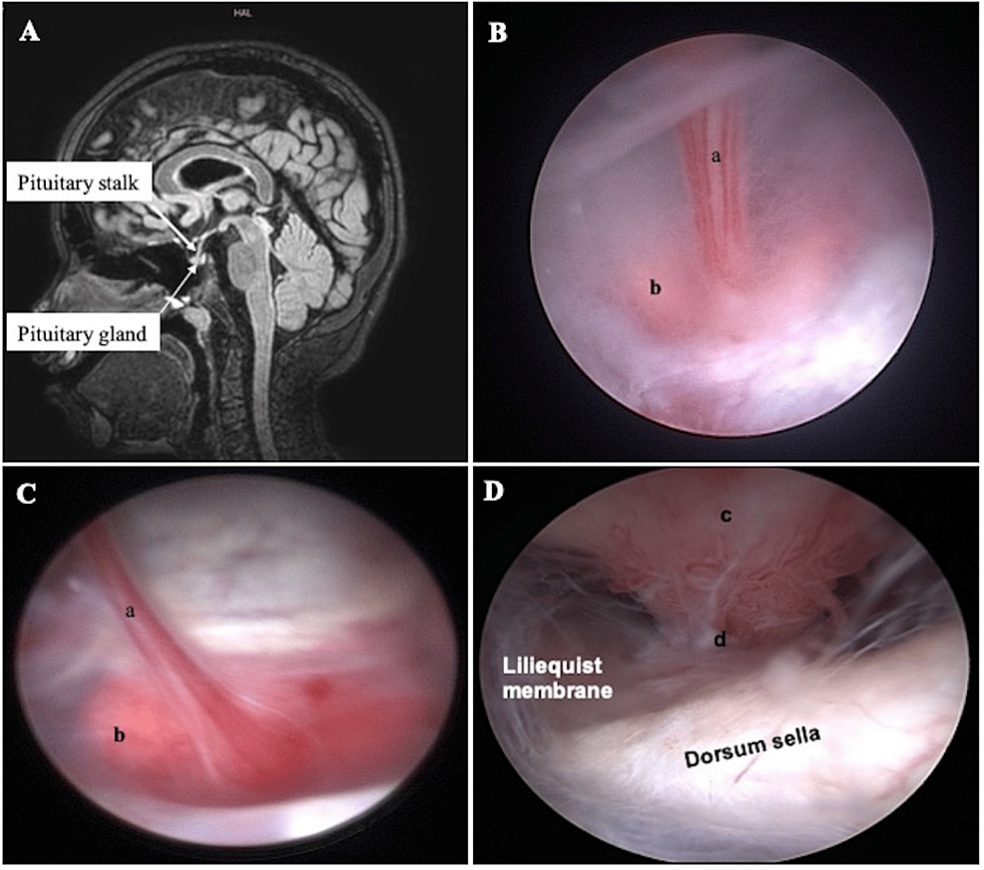
The pituitary stalk and gland. (A) Sagittal T1-weighted brain MRI shows the pituitary stalk and gland that occupies the sella. (B, C) In the endoscopic view, the pituitary stalk is covered with fenestrated capillary loops, and the reddish-grey gland has a richer, smaller vessel (plexus). (D) The dorsum sella is visualized posteriorly, and the area is laterally covered with a Liliequist membrane. a, pituitary stalk; b, pituitary gland; c, tuber cinereum; d, the junction between the stalk and the gland as identified by the beginning of the rich smaller vessel (plexus).

The pituitary gland sits within the sella turcica of the sphenoid bone at the base of the skull. It is a well-vascularized tissue with a dual blood supply from the hypothalamohypophyseal portal system (mainly for the anterior gland) and the inferior hypophyseal artery (posterior gland) [14]. The size of the pituitary gland varies depending on age and gender. In children, Sari et al. [15] reported the median height and volume of 8.48 ± 1.08 mm and 3.91 ± 0.75 mm for girls and a smaller size for boys, i.e., 6.19 ± 0.88 mm and 3.81 ± 0.68 mm. During the neuroendoscopic procedure, a healthy pituitary stalk and gland are seen with fenestrated capillary loops or primary capillary plexus around the stalk. Meanwhile, the secondary capillary plexus can be visualized closer to the gland as rich smaller vessels (Figure 4). Thus, the depiction of the capillary plexus demonstrates the anatomical relationship and transition area of the pituitary stalk and gland.

The Liliequist membrane

**Figure 5:**
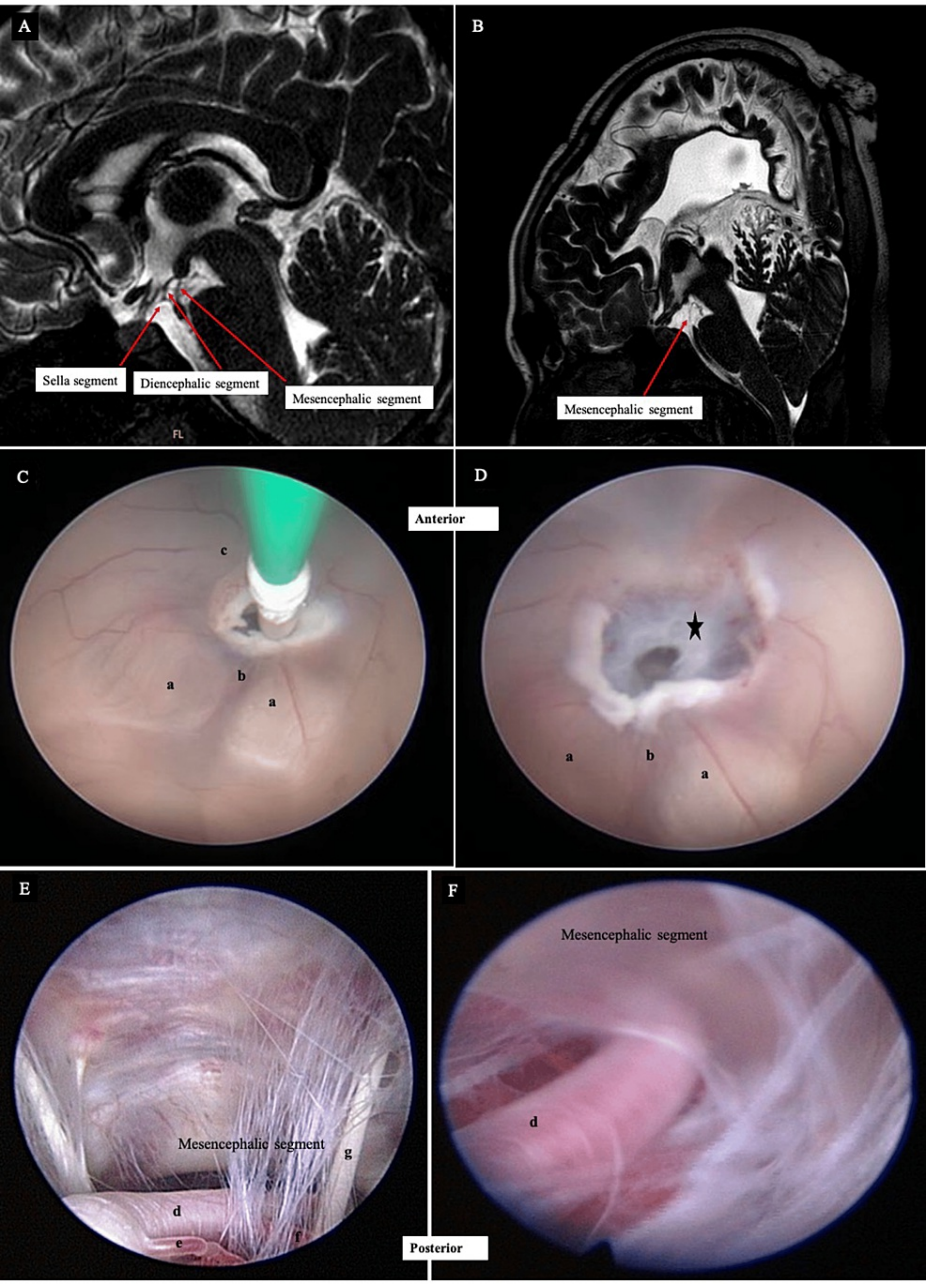
Anatomical images of the Liliequist membrane. (A) Mid-sagittal CSF DRIVE brain MRI of a child shows the segments of the Liliequist membrane. (B) A similar sequence of different children with agenesis of corpus callosum who only have the mesencephalic segment. (C) Neuroendoscopic images were captured during the endoscopic third ventriculostomy procedure in a child with meningitic hydrocephalus. The diencephalic leaf of the Liliequist’s membrane was initially expected to be opened simultaneously with the third ventricular floor during stoma creation. (D) The leaf is found to be a dense sheet-like membrane without openings (star) and requires further coagulation to open the membrane. (E, F) The mesencephalic leaf with variable arachnoid trabeculae and is attached to the oculomotor nerve. a, mammillary bodies; b, basilar artery underneath; c, floor of the third ventricle; d, basilar artery; e and f, perforating basilar artery; g, right oculomotor nerve.

Dias et al. illustrated the anatomy of the Liliequist membrane in relation to imaging findings [16]. The three segments, i.e., the sellar, mesencephalic, and diencephalic, lie beneath the third ventricle, anteriorly extending from the dorsum sellae to the mammillary bodies [17]. The sellar segment is located closer to the dorsum sella (Figure 4D), while the diencephalic segment extends posteriorly. The mesencephalic leaf has previously been reported to have variable arachnoid trabeculae (Figure 5). It is incomplete and thinner and presents a fenestration through which the basilar artery passes. Laterally, the membrane has insertions into the oculomotor nerves or adjacent to them.

An incidental calcified pineal gland

The normal pineal gland appears as a small reddish-brown structure [18]. It is anatomically located outside the blood-brain barrier and has a rich network of blood vessels [19]. The pineal gland or epiphysis cerebri is a mysterious gland in the body thought to play a major role in controlling circadian and seasonal cycles and in regulating melatonin and other hormones.

**Figure 6:**
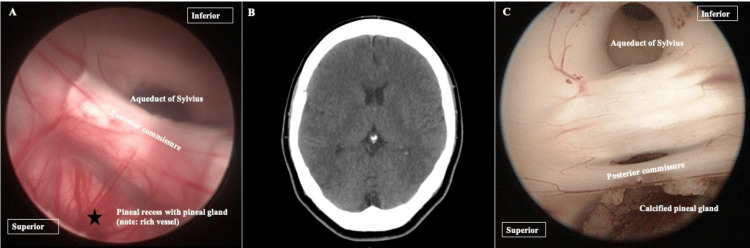
The pineal gland. (A) The neuroendoscopic view shows the pineal recess with a rich network of blood vessels at the area of the pineal gland (star). (B) In a noncontrast CT brain, when there is a presence of calcium, the pineal gland will appear hyperdense (circle). (C) It is uncommon to see the actual features of a calcified pineal gland.

Pineal calcification, identified as hyperdensity in the noncontrast CT brain, might be a physiological process and not associated with pathological or aging changes [20]. Interestingly, during the procedure, a calcified pineal gland (corpora arenacea or brain sand) may be seen. The anatomical changes visualized as a devoid vascularised supply, sand-like appearance with no associated cystic changes or mass within or peripheral to the gland. It is an uncommon view to see the actual features of a calcified pineal gland (Figure 6) [21].

Neuroendoscopic view of hematoma within the aqueduct of Sylvius

**Figure 7:**
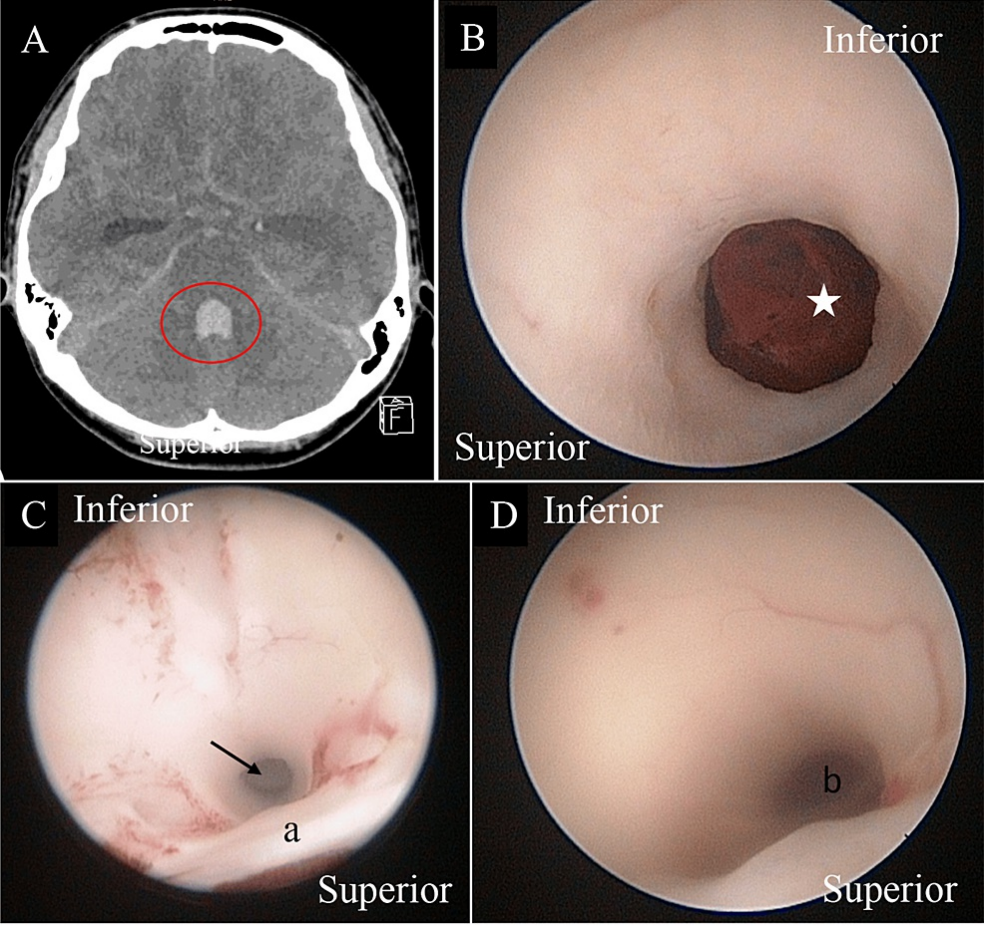
A child presented with sudden onset loss of consciousness with a Glasgow coma score of 6/15. (A) His CT brain disclosed intraventricular hemorrhages, particularly within the fourth ventricle (circle), secondary to rupture arteriovenous malformation. Images taken during the endoscopic washout of the hematomas. (B) Neuroendoscopy image at a closer look shows that the hematoma blocks the aqueduct of Sylvius (star). Ventricular irrigation was able to break down the clot, with (C) blood degradation products causing neuroinflammatory changes surrounding the ventricle. This was then removed successfully with the aqueduct re-establishment (arrow). (D) Another image from different patients shows the normal view of the aqueduct of Sylvius. a, posterior commissure; b, aqueduct of Sylvius.

Intraventricular hemorrhage, an accumulation of blood within the ventricles, can be due to primary or secondary causes [22]. It arises either within the ventricles or via an extension into the ventricle from intracerebral hemorrhage. The volume of blood within the ventricles may influence the patient’s outcome [23]. A concern to this is the development of hydrocephalus, whether non-communicating vs communicating or acute vs chronic. The debris of the blood clots that obstruct the ventricular channel, such as the aqueduct of Sylvius, can lead to non-communicating hydrocephalus. As time progresses, the neuroinflammatory process further damages the ependymal layer of the ventricles, resulting in discontinuity of the surface, gliosis, and scarring [24,25]. One way of preventing this is via an endoscopic washout of the hematoma, which allows the clearing of the blood products that are obstructing the ventricular channel (Figure 7) and, in the future, reduces the permanent VPS dependency [26].

Tetraventricular hydrocephalus with cystic dilatation of the foramen of Magendie and Luschka

**Figure 8:**
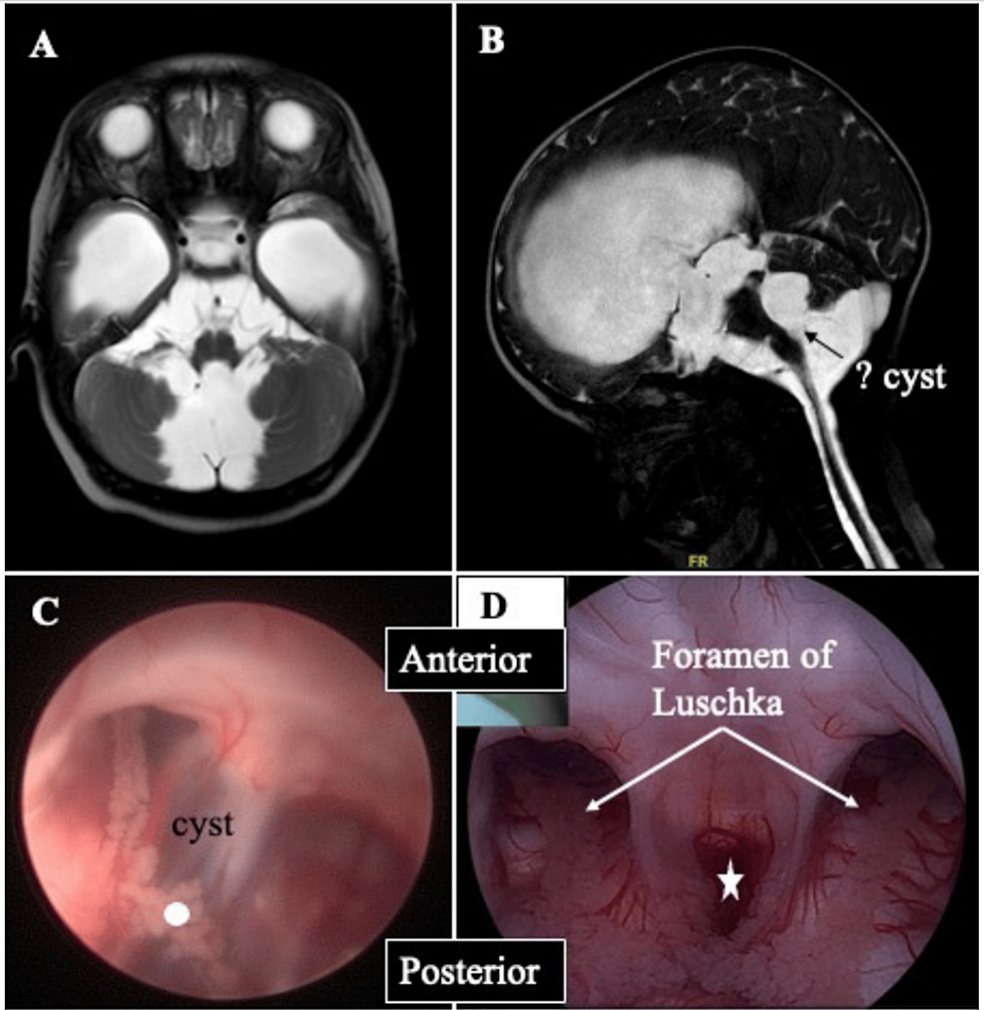
A 14-year-old boy presented with raised intracranial pressure. (A, B) The axial and sagittal T2-weighted brain MRI shows the cystic dilatation of the fourth ventricle, filling the posterior fossa. The image also showed a layer of arachnoid membrane at the lower level of the fourth ventricle. The patient underwent an endoscopic guided fenestration of the cyst. (C) The arachnoid membrane is visualized, and posterior to it is the choroid plexus of the fourth ventricle (circle). (D) Following endoscopic fenestration, CSF patency is established, and the foramen of Magendie (star; midline) and the foramen of Luschka (lateral bilaterally) are visualized.

The patient presented with raised ICP symptoms, and subsequent MRI brain (Figures 8A-8B) shows a cystic dilatation of the fourth ventricle filling the posterior fossa. In cases where the dilatation of the fourth ventricle is so great, it may cause compression of the brainstem against the clivus [27]. The membrane of the cyst may also obstruct the fourth ventricle outlet, causing hydrocephalus. Figures 8C-8D show the radiological findings of the cystic cavity and endoscopic fenestration of the cysts, resulting in the CSF flow patency with the third ventricle. The neuroendoscopic view portrays an interesting image of the foramen of Magendie (midline) and the foramen of Luschka (lateral bilaterally).

## Discussion

The neurosurgical armamentarium has expanded over the last decade with many new products, in particular neuroendoscopic equipment coming out on the market. Together with new technologies, neurosurgeons develop new skills and later become more comfortable with the technique. The practice is widespread in pediatric neurosurgical cases. In this collection, the authors look at different cases and try to appreciate the neuroendoscopic images and correlate them with radiological imaging. For example, in selected cases such as ETV, this avoids the use of CSF diversion such as permanent ventriculoperitoneal shunt or temporary external ventricular drain to treat hydrocephalus [26]. Some cases are considered straightforward but some require a great understanding of underlying clinical history. In clinical cases such as a child with ventriculitis (Figure 3), the thickened floor of the third ventricle is well visualized during the ETV procedure, even though the radiological image cannot be appreciated. The leaflets of the Liliequist membrane (Figure 5) also vary, and during the formation of a stoma, the membrane may or may not adhere to the floor of the ventricle [16].

Familiarity with neuroendoscopy and getting comfortable with viewing via an endoscopic camera are important considering that, once within the ventricle, the surgeon has to carefully navigate the scope to prevent injury to the neurovascular bundle. Once the normal anatomy is lost, such as in intraventricular hemorrhage (Figure 7), clearing the view via irrigation helps. Furthermore, having neuronavigation may help ease the burden of going into the wrong ventricle or failure to identify the region of interest. Moreover, in patients with a loculated cystic cavity (such as in Figure 8), the surgeon must know the 360 views of the structure surrounding the membrane prior to fenestration. Thermal damage caused by bipolar electrocoagulation or suboptimal location of cyst fenestration that inadvertently injures the blood vessel are avoidable complications.

As the authors share these collective case series, the pitfalls and drawbacks of using neuroendoscopy are perhaps the maneuvering of the endoscope that is within a small space while respecting the adjacent normal neurovascular area. Within the ventricular cavity, the surgeons may easily get lost, mainly due to the distorted normal anatomy or configuration of the ventricle. Appreciating certain anatomical landmarks, such as mammillary bodies that serve as the posterior landmarks for ETV or pineal glands that serve as superior and posterior landmarks to the aqueduct of Sylvius, is crucial. Other limitations include the availability of follow-up imaging, particularly MRI brain to confirm whether the intraoperative procedure was successful. Variations of patients' pathology may require further collective series, and hopefully, in the future, there will be an interest in other centers to share their series.

## Conclusions

These neuroendoscopic images are part of the team’s collective experiences in delivering treatment for pediatric patients. Together with the existing knowledge of radiological images, the feasibility and effectiveness of the neuroendoscopic technique help surgeons appreciate the anatomy. In the future, expansion of the neurosurgical treatment via neuroendoscopy alone or together with craniotomy will maximize patient care.
